# Retraction Note to: BRD4 promotes tumor progression and NF-κB/CCL2-dependent tumor-associated macrophage recruitment in GIST

**DOI:** 10.1038/s41419-025-07520-4

**Published:** 2025-03-24

**Authors:** Jianfeng Mu, Pengfei Sun, Zhiming Ma, Pengda Sun

**Affiliations:** 1 Department of Gastric and Colorectal Surgery, The First Hospital of Jilin University, Changchun, Jilin Province China; 2 Changchun Railway Medical Insurance Management Office, Changchun, Jilin Province China; 3 Department of Gastrointestinal Nutrition and Hernia Surgery, The Second Hospital of Jilin University, Changchun, Jilin Province China

Retraction Note to: *Cell Death and Disease* 10.1038/s41419-019-2170-4, published online 09 December 2019

The Editors-in-Chief have retracted this article because of concerns regarding the figures presented in this work. These concerns call into question the article’s overall scientific soundness. An investigation conducted after its publication discovered the following issues:

- Panels *Normal, Case 1* and *Tumor, Case 1* in Figure 1C appear to overlap, when rotated, with Panels *H716* and *CO802 D5* in Fig. 6 in [1];
- Panel *Vector, GIST-T1* in Figure 2E appears to overlap, when rotated, with Panel *mir-9-5p mimics control*, *MKN-45* in Fig. 3B in [2];
- Panel *Vector, GIST-882* in Figure 2E appears to overlap, when rotated, with Panel *HEC1A*, *si-TTB-AS1+miR-NC* in Fig. 4E in [3];
- Panel *BRD4* in Figure 3E appears to overlap, when rotated, with the first panel of Fig. 3A in [4];
- Panels *Vector, BRD4* and *BRD4, BRD4* in Figure 4C appear to overlap, when rotated, with Panels C and E with Fig. 4 in [5];
- The four panels in Figure 7A appear to overlap, when rotated, with Panels *24* *h, BRAF V600E*, *24* *h, pEGFR*, and *24* *h, EGFR* in Fig. 3 and *Ischemia, BRAF V600E* in Fig. 4 in [5];
- The two panels in Figure 3F appear to overlap, when rotated, with second and third panels of Fig. 3A in [6].

The panels in question represent tissues taken from animals subject to different experimental conditions. The Editors-in-Chief therefore no longer have confidence in the integrity of the research presented in this article.

The authors have not replied to correspondence from the Publisher about this retraction.
